# Establishment and validation of a prediction model for nonrecovery of left ventricular ejection fraction in acute myocardial infarction patients combined with decreased left ventricular ejection fraction

**DOI:** 10.1002/clc.24212

**Published:** 2024-01-29

**Authors:** Yang Yang, You Zheng Dong, An Xue Hou, De Ping Liu, Jin Wu He, Jun Ye Chen, Xing Hua Jiang

**Affiliations:** ^1^ Department of Cardiology The Second Affiliated Hospital of Nanchang University Nanchang Jiangxi China

**Keywords:** acute myocardial infarction, left ventricular ejection fraction, nomogram, percutaneous coronary intervention, prediction model

## Abstract

**Background:**

This study aimed to investigate the risk factors for nonrecovery of left ventricular ejection fraction (LVEF) during follow‐up in patients with acute myocardial infarction (AMI) who underwent percutaneous coronary intervention (PCI) combined with reduced LVEF, and establish and verify a risk prediction model based on these factors.

**Methods:**

In this study, patients with AMI who underwent PCI in a high‐volume PCI center between December 2018 and December 2021 were consecutively enrolled, screened, and randomly assigned to the model establishment and validation cohorts. A predictive model method based on least absolute shrinkage and selection operator regression was used for establishment and validation.

**Results:**

Cardiac troponin I, myoglobin, left ventricular end‐diastolic dimension, multivessel disease, and no‐reflow were identified as potential predictors of LVEF recovery failure. The areas under the curve were 0.703 and 0.665 in the model establishment and validation cohorts, respectively, proving that the prediction model had some predictive ability. The calibration curves of the two cohorts showed good agreement with those of the nomogram model. In addition, the decision curve analysis showed that the model had a net clinical benefit.

**Conclusion:**

This prediction model can assess the risk of nonrecovery of LVEF in patients with AMI undergoing PCI combined with LVEF reduction during follow‐up, and conveniently screen high‐risk patients with nonrecoverable LVEF early.

## Graphical abstract

Establish and validate a predictive model based on patients with acute myocardial infarction treated with percutaneous coronary intervention, whose left ventricular ejection fraction decreased but did not recover during follow‐up.

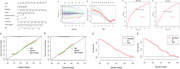

## 1 INTRODUCTION

Acute myocardial infarction (AMI) is a severe subtype of coronary atherosclerosis.^1^ Although revascularization strategies such as percutaneous coronary intervention (PCI) and drugs have made significant progress in recent years, ventricular remodeling and recovery of cardiac function after AMI remain the main factors determining the long‐term prognosis of patients with AMI.^2^ A change in left ventricular ejection fraction (LVEF) is an essential manifestation of ventricular remodeling.^3^

Compared with other diseases that cause heart failure (HF), such as dilated cardiomyopathy and rheumatic heart disease, many risk factors for AMI can be reversed in the acute phase, improving the LVEF of patients with AMI and preventing HF. Acute anterior wall myocardial infarction and a history of AMI are essential factors affecting LVEF.^4^ Some studies^5^, ^6^ have shown that high troponin levels represent large infarct size, which can identify patients with LVEF < 40%, and myoglobin is also closely related to infarct size, and 65% of patients with HF after AMI have myoglobin (Mb) > 800 ng/mL.^7^ In addition, left ventricular end‐diastolic dimension (LVEDD),^8^ the number of vascular lesions^9^, ^10^ and no‐reflow.^11^ were independent risk factors for HF in AMI patients.

These studies suggest that early interventions can improve LVEF for a long time in patients with AMI and reduced LVEF. Information on a single predictor is usually insufficient to provide a reliable diagnosis or risk estimation. In contrast, the prediction model associates multiple predictors with the probability or risk of diagnosis or prognosis, which can more accurately screen patients and better assist physicians in disease prevention and treatment.^12^ Lei et al.^13^ demonstrated that a low troponin T peak, a nonanterior myocardial infarction, and low heart rate could be combined to predict LVEF recovery. However, this study only focused on LVEF recovery in patients with ST‐segment elevation myocardial infarction, and there is currently no research on predicting the nonrecovery of LVEF in AMI patients. Therefore, this study aimed to investigate the risk factors for poor recovery or a persistent decline in LVEF during follow‐up in AMI patients, combined with reduced LVEF (≤50%) undergoing PCI, to establish and validate a risk prediction model based on these factors, and to provide a direction for clinical diagnosis and treatment.

## 2 STUDY MATERIALS AND METHODS

### 2.1 Population and study design

This was a single‐center observational study of the medical record system of the Second Affiliated Hospital of Nanchang University from December 2018 to December 2021. Patients with AMI selected for PCI were consecutively screened according to the inclusion and exclusion criteria, and randomly divided into the model establishment and validation cohorts in a 7:3 ratio. In addition to fulfilling the diagnostic criteria for AMI^14^ and PCI,^15^, ^16^ the patients included in this study also underwent their first echocardiographic examination (admission ≤48 hours) during hospitalization, which showed LVEF ≤ 50% and had ≥1 LVEF value during follow‐up. Patients were not eligible for inclusion if they met the following exclusion criteria: (1) age <18 years; (2) history of HF and had been identified as having HF caused by rheumatic heart disease, dilated cardiomyopathy, or other structural heart diseases; (3) and other comorbidities including severe liver and kidney dysfunction, blood system disorders, or acute cerebrovascular disease.

### 2.2 Data collection

We reviewed the previous literature in detail^4^, ^9^, ^10^, ^17^, ^18^, ^19^ and combined it with the clinical data available at the center to collect the following clinical variables: baseline patient data, first laboratory test data on admission, various examination data, PCI surgical status, discharge medication, and LVEF values during follow‐up.

### 2.3 Group definition

Patients were followed up within 1, 3, 6, and 12 months after surgery to obtain LVEF values. As long as ≥1 LVEF value was present during the follow‐up period, we took the LVEF value at the last follow‐up as the target LVEF value during the follow‐up period. Patients were divided into two groups based on the LVEF difference value (between the target LVEF during follow‐up and the baseline LVEF) (△LVEF). That was, the good LVEF recovery group with △LVEF > 5%, and the nonrecovery of the LVEF group included poor LVEF recovery (△LVEF ≤ 5%) or decreased LVEF (LVEF continued to decline over time).^20^, ^21^

### 2.4 Sample size

The variables must have at least 10 events to avoid overfitting during model building.^22^ Based on these rules, there were 174 events in this study: 122 in the model establishment cohort and 52 in the model validation cohort. This study had a sufficient sample size to consider 12 variables as predictive factors for establishing a model.

### 2.5 Statistical analyses

Before analyzing the data, we checked all variables' distribution and missing values. However, no apparent abnormal distribution was found in all variables, and the missing value of variables was less than 5%. The chain equations in the “mice” package of the R language software were used to perform 10‐fold multiple interpolations for missing data.^23^

The R language version 4.2.2 software* and SPSS version 25.0 software were used for analysis, and the Kolmogorov–Smirnov test was used to test for normal distribution. Continuous variables were expressed as mean ± standard deviation or median (interquartile interval), and group comparisons were made using the *t*‐test or Mann–Whitney *U* test. Classification variables were expressed as percentages, and comparisons between the groups were made using the *χ* ^2^ or Fisher's exact test.

The “kappa” and “cor” functions in the R language software were used to compute the eigenvalues to make a judgment on multicollinearity before the least absolute shrinkage and selection operator (LASSO) regression.^24^ The LASSO regression was used to evaluate 47 candidate variables in the established model cohort, and 10‐fold cross‐validation was conducted to screen out statistically significant potential predictive variables.^25^ Multivariate logistic regression analysis was performed to screen the clinical variables used to construct the predictive model. The consistency indices (C‐indices) of established and validated model cohorts were calculated. The accuracy of the model was determined using the areas under the curve (AUC) of the subjects' receiver operating characteristic curve (ROC). A calibration curve was used to assess the degree of calibration,^26^ and the decision curve analysis (DCA) was performed to determine the clinical utility of the prediction model.^27^ All tests were conducted on both sides, with inspection level *α* = .05. The establishment and validation of the new nomogram were based on the guidelines for transparent reporting of a multivariate prediction model for individual prognosis or diagnosis.^12^

## 3 RESULTS

### 3.1 Analysis of patient characteristics for model establishment cohort and model validation cohort

**Figure 1:**
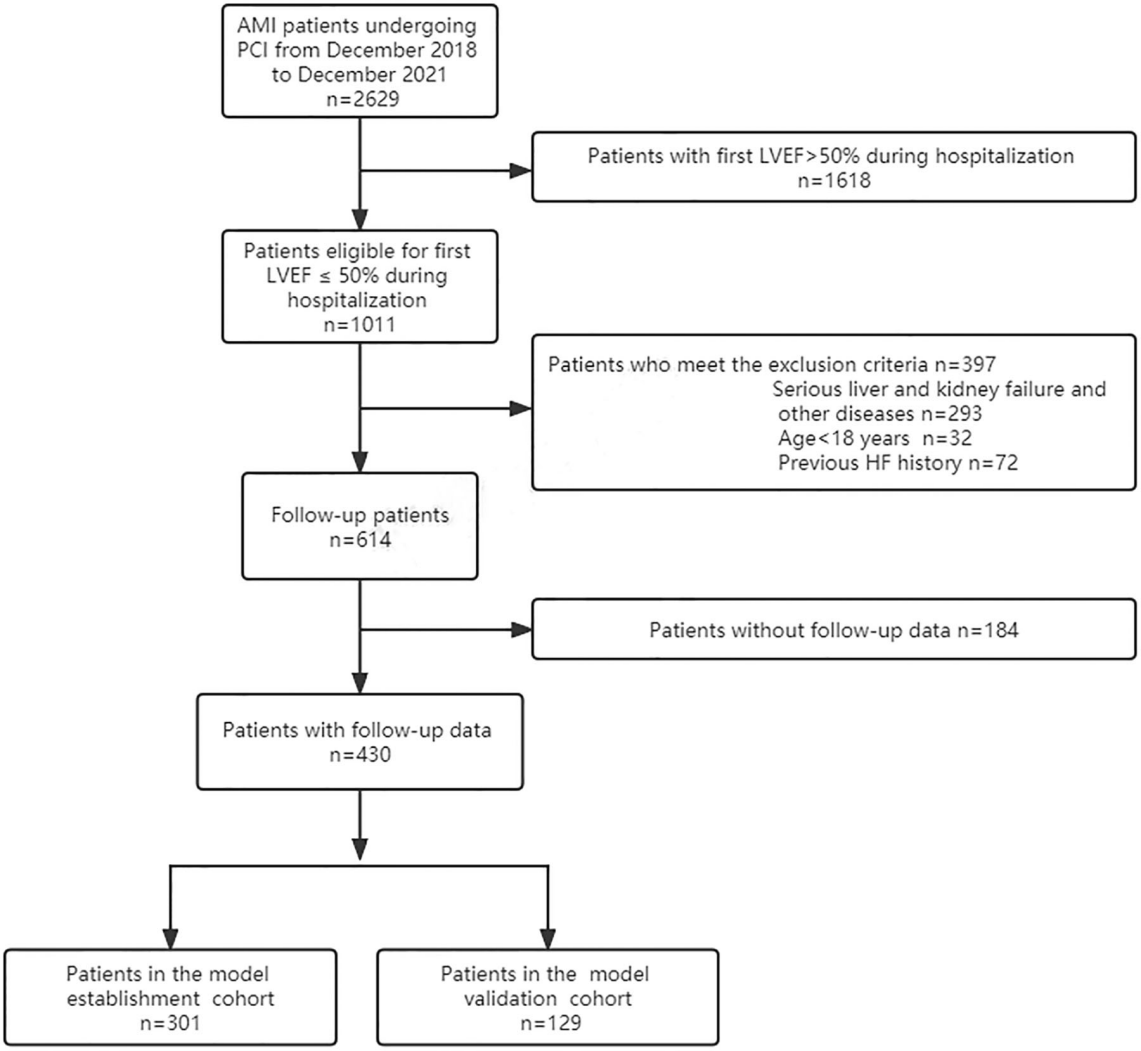
Study cohort flow diagram. AMI, acute myocardial infarction; LVEF, left ventricular ejection fraction; PCI, percutaneous coronary intervention.

A total of 2629 patients with AMI who underwent PCI were enrolled, and 430 were selected for inclusion in the total cohort (Figure 1). The median follow‐up duration was 6 (3–12) months. In the overall cohort, 301 and 129 patients were randomly assigned to the model establishment and validation cohorts. There were no statistically significant differences in the clinical characteristics of patients in the model establishment and validation cohorts (all *p* > .05) (Table 1). The rates of LVEF nonrecovery during follow‐up were 40.53% (122/301) and 40.31% (52/129) in both cohorts, respectively.

**Table 1:** Patients characteristics of the model establishment cohort and model validation cohort.

Variables	Model establishment cohort (*n* = 301)	Model validation cohort (*n* = 129)	*p*‐Value
Baseline data			
Age (years)	68 (59, 75)	68 (59, 77)	.837
Male, *n* (%)	242 (80.40)	107 (82.95)	.628
Systolic blood pressure (mmHg)	120 (106, 136)	123 (106, 139)	.227
Diastolic blood pressure (mmHg)	76 (66, 84)	77 (67, 85)	.584
Heart rate (beats/min)	86 (75, 96)	83 (75, 95)	.587
Killip grade ≥III, *n* (%)	160 (53.16)	74 (57.36)	.486
Previous medical history			
Diabetes, *n* (%)	82 (27.24)	42 (32.56)	.318
Hypertension, *n* (%)	162 (53.82)	63 (48.84)	.399
Cerebrovascular disease, *n* (%)	39 (12.96)	19 (14.73)	.735
Smoking, *n* (%)	126 (41.48)	50 (38.76)	.623
Drinking, *n* (%)	54 (17.94)	27 (20.93)	.554
Myocardial infarction, *n* (%)	29 (9.63)	20 (15.50)	.112
PCI or CABG, *n* (%)	22 (7.31)	17 (13.18)	.079
Atrial fibrillation, *n* (%)	18 (5.98)	8 (6.20)	1
Laboratory testing			
WBC (×10^9^/L)	9.23 (6.97, 12.01)	8.61 (6.23, 11.25)	.148
PLT (×10^9^/L)	204 (163, 254)	204 (160, 239)	.562
Hb (g/L)	136 (122, 148)	132 (119, 147)	.402
GHB (%)	5.90 (5.60, 6.7)	5.90 (5.60, 7)	.470
BNP (pg/mL)	473.80 (186.47, 964.19)	473.80 (199.01, 921.67)	.997
Ua (μmol/L)	398.76 (330.82, 474.63)	390.09 (315.23, 473.15)	.439
Scr (μmol/L)	87.20 (72.08, 106.82)	82.56 (71.60, 104.24)	.486
CK‐MB (U/L)	37.21 (20.34, 93.17)	35.25 (21.20, 87.48)	.903
cTnI (ng/mL)	12.44 (3.13, 49.45)	12.44 (3.08, 50)	.837
Mb (ng/mL)	157.98 (67.27, 587.64)	173.46 (70.79, 542.18)	.837
TC (mmol/L)	4.49 (3.83, 5.25)	4.44 (3.65, 4.85)	.131
TG (mmol/L)	1.28 (0.99, 1.73)	1.24 (1.02, 1.7)	.695
LDL‐C (mmol/L)	2.70 (2.21, 3.35)	2.70 (2.09, 3.13)	.268
LP‐a (mg/L)	25.42 (12.66, 47.83)	25.42 (12.19, 78.31)	.379
Various inspections			
LVEF (%)	44 (40, 47)	44 (40, 47)	.546
LVESD (mm)	39 (36, 43)	38 (36, 43)	.664
LVEDD (mm)	50 (46, 55)	49 (46, 55)	.847
STEMI, *n* (%)	229 (76.08)	102 (79.07)	.582
Anterior wall myocardial infarction, *n* (%)	154 (51.16)	64 (49.61)	.850
Operation status			
TIMI blood flow level 0–1 before operation, *n* (%)	194 (64.45)	77 (59.69)	.407
Left main stem disease, *n* (%)	37 (12.29)	21 (16.28)	.340
Multivessel disease, *n* (%)	234 (77.74)	106 (82.17)	.365
Multiple stents, *n* (%)	166 (55.15)	66 (51.16)	.513
Total diameter of stents (mm)	5.50 (3, 7.50)	4.75 (3, 7.50)	.427
Total length of stents (mm)	48 (29, 72)	38 (29, 69)	.564
Intraoperative use of tirofiban, *n* (%)	21 (6.98)	13 (10.08)	.370
No‐reflow, *n* (%)	27 (8.97)	12 (9.30)	1
Gensini score, points	72 (42, 90)	68 (40, 86)	.705
Discharge medication			
Antiplatelet drugs, *n* (%)	285 (94.68)	124 (96.12)	.696
Statins, *n* (%)	281 (93.36)	117 (90.70)	.446
β‐Receptor blockers, *n* (%)	286 (95.02)	121 (93.80)	.779
ACEI or ARB drugs, *n* (%)	177 (58.80)	81 (62.79)	.505
Diuretics, *n* (%)	196 (65.12)	78 (60.47)	.418
Unrecovered LVEF	122 (40.53)	52 (40.31)	1

Abbreviations: ACEI, angiotensin‐converting enzyme inhibitor; ARB, angiotensin receptor antagonist; BNP, brain natriuretic peptide; CABG, coronary artery bypass grafting; CHB, glycated hemoglobin; CK‐MB, creatine kinase isoenzyme; CTnI, troponin I; Hb, hemoglobin; LDL‐C, low‐density lipoprotein cholesterol; LP‐a, lipoprotein a; LVEDD, left ventricular end‐diastolic dimension; LVEF, left ventricular ejection fraction; LVESD, left ventricular end‐systolic dimension; Mb, myoglobin; PCI, percutaneous coronary intervention; PLT, platelet; Scr, serum creatinine; STEMI, ST‐segment elevation myocardial infarction; TC, total cholesterol; TG, triglyceride; TIMI, thrombolysis for myocardial infarction before treatment; UA, uric acid; WBC, white blood cells.

### 3.2 Filter prediction variables for unrecovered LVEF

**Figure 2:**
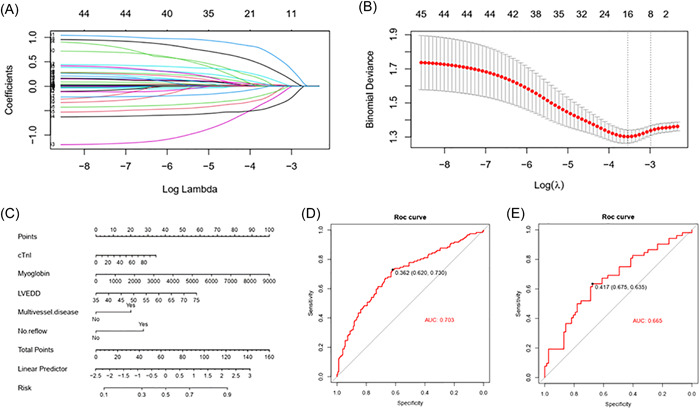
(A) The distribution of LASSO regression coefficients for 47 variables. (B) Penalty plot for the LASSO model. (C) A nomogram of nonrecovery of LVEF. (D) ROC curve of model establishment cohort. (E) ROC curve of model validation cohort. AUC, areas under the curve; cTnI, troponin I; LASSO, least absolute shrinkage and selection operator; LVEDD, left ventricular end‐diastolic dimension; LVEF, left ventricular ejection fraction; ROC, receiver operating characteristic curve.

Collinearity diagnostic test indicated significant collinearity between candidate variables included in this study and the ratio of the maximum and minimum eigenvalues was 136.066. Therefore, LASSO regression analysis was used to simplify and screen the 47 research variables of the model establishment cohort to select the predictive variables for unrecovered LVEF. Using a 10‐fold cross‐validation method, the Lambda.1se value with the smallest validation error was used as the optimal solution for the screening model, and variables with nonzero coefficients were statistically analyzed. After LASSO regression analysis, 47 variables were reduced to five potential prediction variables with nonzero coefficients, including troponin I (CTnI), Mb, LVEDD, multivessel disease, and no‐reflow (Figure 2A,B).

### 3.3 Construction of nomogram

The variables selected from the LASSO regression were analyzed using univariate and multivariate logistic regression. cTnI, Mb, LVEDD, multivessel disease, and no‐reflow were independent risk factors for LVEF recovery (all *p* < .05) (Table 2). A nomogram was constructed based on these five variables (Figure 2C). The total risk score formula of the nomogram was *Y* = 0.346 × cTnI + 0.011 × Mb + 1.450 × LVEDD + (−50.742) + 20.113 × multivessel disease + 27.376 × no‐reflow. The linear probability formula was LP = 0.039 × *Y* + (−2.485). The probability formula was *p* = −7.560 × 10^−7^ × *Y* ^3^ + 0.0001 × *Y* ^2^ + 7.681 × 10^−5^ × *Y* + 0.092, where the units of cTnI, Mb, LVEDD was ng/mL, ng/mL, mm, multivessel disease was one (nonmultivessel disease was zero), and no‐reflow was one (reflow was zero).

**Table 2:** Predictors of nonrecovery of LVEF.

Variables and intercept	Univariate logistic regression				Multivariate logistic regression			
	*β*	OR	95% CI	*p*‐Value	*β*	OR	95% CI	*p*‐Value
cTnI	.014	1.014	1.003–1.025	.016	.013	1.013	1–1.026	.036
Mb	<.001	1	1–1.001	.002	<.001	1	1–1.001	.008
LVEDD	.041	1.042	1.007–1.079	.018	.056	1.058	1.020–1.096	.003
Multivessel disease	.686	1.986	1.116–3.655	.023	.777	2.174	1.180–4.145	.015
No‐reflow	1.007	2.736	1.226–6.414	.016	1.057	2.877	1.240–6.991	.016
Intercept	NA	NA	NA	NA	−4.445	0.012	0.001–0.086	<.001

Abbreviations: *β*, regression coefficient; CI, confidence interval; cTnI, troponin I; LVEDD, left ventricular end‐diastolic dimension; LVEF, left ventricular ejection fraction; Mb, myoglobin; NA, not available; OR, odds ratio.

### 3.4 Verification of nomogram

**Figure 3:**
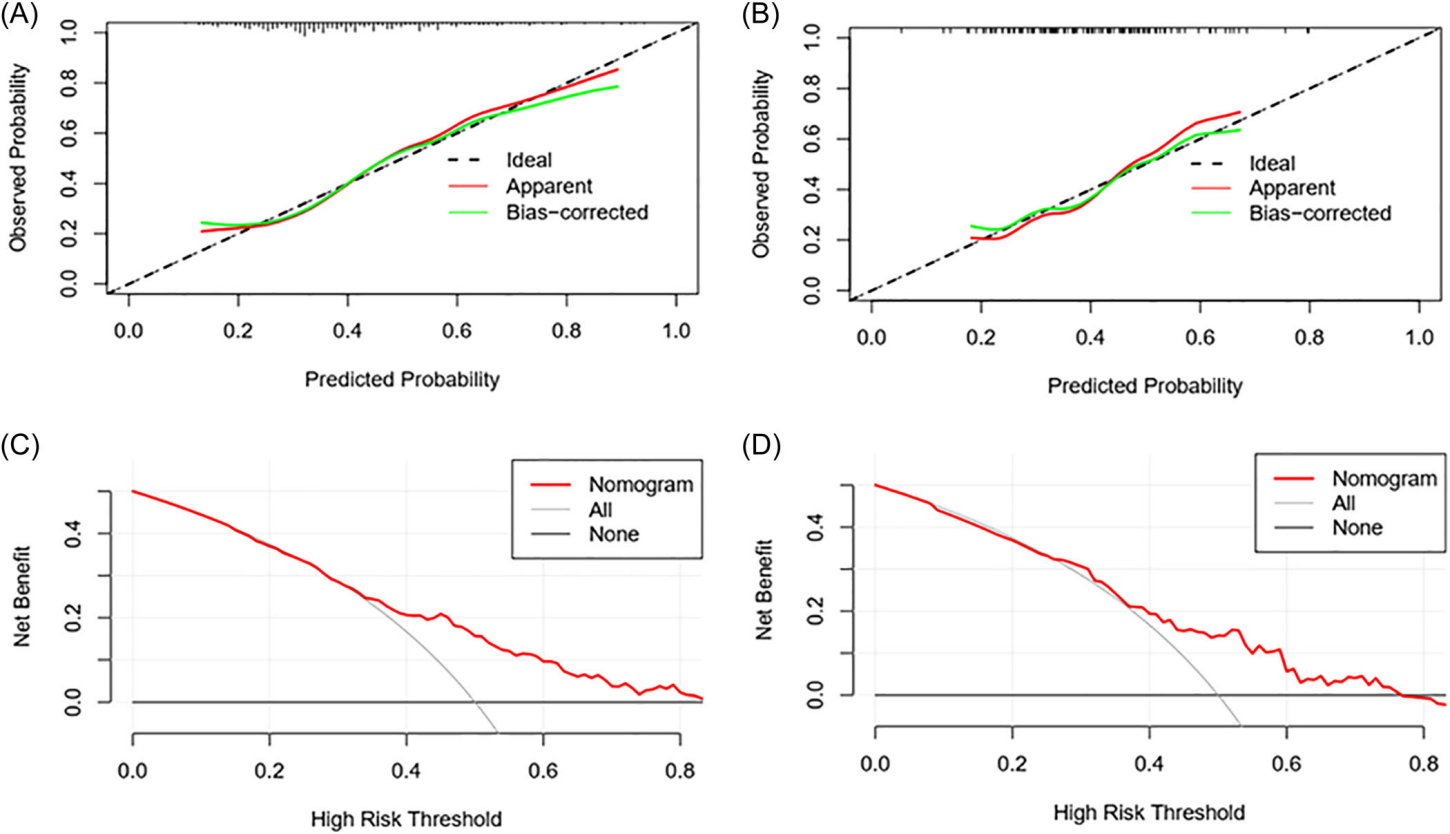
(A) Calibration curve for the model establishment cohort. (B) Calibration curve for the model validation cohort. (C) DCA curve for the model establishment cohort. (D) DCA curve for the model validation cohort. DCA, decision curve analysis.

The nomogram was internally validated by measuring the model's discrimination, calibration, and clinical utility in the establishment and validation cohorts. The C‐index of the model establishment cohort was 0.703 (0.642–0.764), and the model validation cohort was 0.665 (0.568–0.762). On the ROC curve, the AUC of the model establishment cohort (Figure 2D) was 0.703, and the AUC area of the model validation cohort (Figure 2E) was 0.665. The calibration curve showed strong agreement between the model constructed using the nomogram and the ideal model (Figure 3A,B). The Hosmer–Lemeshow test showed that the logistic regression model was consistent with the data (model establishment cohort, *p* = .858 > .05; model validation cohort, *p* = .872 > .05). The above results demonstrated that the nomogram could effectively predict the nonrecovery of LVEF.

### 3.5 Clinical application of the nomogram

The DCA of the model establishment cohort showed that when the threshold was between 0.25 and 0.84, additional net benefits could be provided (Figure 3C), and the DCA of the model validation cohort showed that the threshold of additional net benefits could be provided in the range of 0.23–0.80 (Figure 3D). This nomogram could be used to predict the risk of unrecovered LVEF with high accuracy, and may have some significance in clinical applications.

## 4 DISCUSSION

In daily clinical practice, a decrease in LVEF may still occur in patients with AMI, even after PCI and other timely revascularization strategies. In this study, we found that five variables—cTnI, Mb, LVEDD, multivessel disease, and no‐reflow—could independently predict the occurrence of nonrecovery of LVEF. A new predictive model based on these risk factors was also established and validated. The results showed that the predictive tool presented in the nomogram had strong discriminative power, with an AUC of 0.703. The calibration curve showed good agreement between the actual and predicted probabilities in the model establishment and validation cohorts. The DCA curve also showed an association with a high net clinical benefit.

Ventricular remodeling is common in patients with AMI. A decrease in LVEF often represents a severe reduction in cardiac reserve function, which is not only associated with high mortality but also tends to increase early and long‐term cardiovascular risk.^28^ Several studies^9^, ^29^ have shown that 30%–60% of AMI patients with reduced LVEF can gradually improve. This also means that LVEF did not recover or even declined further in some patients with AMI, which is consistent with the findings of this study, which found that 40.47% (174/430) of patients with decreased LVEF did not recover during follow‐up. Traditional risk prediction models, such as the Global Registry of Acute Coronary Events (GRACE) and thrombolysis in myocardial infarction scores, have been widely used to predict the prognosis of patients with AMI.^30^, ^31^ No overlap was found between the model established in this study and the relevant variables included in the GRACE score, and no correlation was found between the Gensini score and LVEF recovery in the LASSO analysis. Moreover, the model did not have some standard variables, such as Killip grade, systolic blood pressure, heart rate, anterior myocardial infarction, and left central disease. A possible reason for this is that the LASSO regression analysis can estimate parameters in high‐dimensional regression, eliminate collinear variables, and screen variables that can improve the model's prediction accuracy. In addition, the complexity of the included patients and candidate variables may have also led to differences in individual predictors between different studies.

In this study, cTnI level was a consistent and independent predictor of LVEF failure during recovery in patients with reduced LVEF after infarction. Studies have shown a negative correlation between cTnI levels and LVEF (*r* = −.5394, *p* = .001); the higher the cTnI level, the lower the LVEF.^32^ Furthermore, the cTnI within 24–48 hours after myocardial infarction was associated with no recovery of LVEF during the 4‐month follow‐up.^33^ cTnI is a sensitive and specific marker of myocardial cell injury. As a classical marker of myocardial injury, cTnI should be included in the nomogram model to predict nonrecovery of LVEF. In contrast to myocardial enzymes, such as troponin, Mb levels increase significantly in the early phase after myocardial infarction, and different studies have confirmed its essential role in disease diagnosis and evaluation.^34^, ^35^ The serum Mb level can be used as an indicator to estimate infarct size within 36 hours of symptom onset.^36^ Delanghe et al.^37^ found that the cumulative release value of Mb was correlated with LVEF (*r* = .513). Multivariate logistic regression analysis performed in this study also showed a significant correlation between Mb and nonrecovery of LVEF (odds ratio = 1, 95% confidence interval: 1–1.001, *p* = .008).

This study also showed that LVEDD could predict the nonrecovery of LVEF, and some studies have found that LVEDD on admission is a risk factor for HF in AMI.^38^ AMI causes fibrotic scarring and myocardial remodeling, leading to an increase in LVEDD and left ventricular end‐diastolic pressure (LVEDP). With a sustained increase in LVEDP, pulmonary artery pressure increases due to escape arrhythmia, ultimately leading to changes in cardiac function indicators, such as LVEF.^39^ This may explain why LVEDD leads to LVEF failure during recovery.

Approximately 50% of patients have multivessel disease,^40^ which represents more extensive coronary artery disease and decreased collateral blood flow, and strongly predicts decreased baseline left ventricular function.^10^ Interestingly, adding one diseased vessel was associated with a 16% reduction in LVEF,^41^ and single‐vessel disease was a significant predictor of left ventricular improvement at 6 months after PCI.^10^ No‐reflow often occurs during PCI for AMI. Studies have shown that the no‐reflow phenomenon is associated with a decrease in LVEF, and that the LVEF, peak filling rate, and peak ejection rate of the no‐reflow group were significantly lower than those of the reflow group.^42^, ^43^ Furthermore, Ndrepepa et al.^44^ found that the LVEF at 6 months after PCI was 47.7 ± 13.1% in the no‐reflow group and 54.2 ± 13.9% in the reflow group (*p* < .001), and that no‐reflow was associated with increased mortality within 1 year.

## 5 LIMITATIONS OF THE STUDY

This study had several limitations. First, the sample size collected in this study was small. Therefore, it is necessary to conduct a large‐sample study to verify the conclusions of this study further. Second, although this study implemented strict inclusion and exclusion criteria, a potential selection bias was inevitable. Third, although many variables were collected in this study, some variables with potential predictors were not analyzed or collected because of a lack of hospital data or collinearity between variables, such as body mass index, left main artery disease, and estimated glomerular filtration rate. Finally, this was a single‐center retrospective study that was not externally validated, and this nomogram model requires external validation and universal evaluation of data from other centers.

## 6 CONCLUSIONS

This study established and validated a personalized nomogram to predict unrecovered LVEF in patients with AMI undergoing PCI with reduced LVEF undergoing PCI. The nomogram contained five readily available and commonly used clinical variables: cTnI, Mb, LVEDD, multivessel disease, and no‐reflow. Although this predictive model provides clinicians with a convenient and accurate clinical tool, further external validation is required. We hope this prediction model can identify and screen high‐risk patients early, and effectively reduce the incidence of unrecovered LVEF in patients with AMI and decreased LVEF.

## Data Availability

The data that support the findings of this study are available from the corresponding author upon reasonable request.

## ENDNOTE

*
https://www.r-project.org

## References

[1] Wright RS , Anderson JL , Adams CD , et al. 2011 ACCF/AHA focused update incorporated into the ACC/AHA 2007 guidelines for the management of patients with unstable angina/non‐ST‐elevation myocardial infarction: a report of the American College of Cardiology Foundation/American Heart Association Task Force on practice guidelines developed in collaboration with the American Academy of Family Physicians, Society for Cardiovascular Angiography and Interventions, and the Society of Thoracic Surgeons. J Am Coll Cardiol. 2011;57(19):215‐367. 10.1016/j.jacc.2011.02.011 21545940

[2] Pfeffer MA , Braunwald E . Ventricular remodeling after myocardial infarction. Experimental observations and clinical implications. Circulation. 1990;81(4):1161‐1172. 2138525 10.1161/01.cir.81.4.1161

[3] Cohn JN , Ferrari R , Sharpe N . Cardiac remodeling—concepts and clinical implications: a consensus paper from an international forum on cardiac remodeling. J Am Coll Cardiol. 2000;35(3):569‐582. 10716457 10.1016/s0735-1097(99)00630-0

[4] McNamara RF , Carleen E , Moss AJ . Estimating left ventricular ejection fraction after myocardial infarction by various clinical parameters. Am J Cardiol. 1988;62(4):192‐196. 3400597 10.1016/0002-9149(88)90210-x

[5] Steen H , Giannitsis E , Futterer S , Merten C , Juenger C , Katus HA . Cardiac troponin T at 96 hours after acute myocardial infarction correlates with infarct size and cardiac function. J Am Coll Cardiol. 2006;48(11):2192‐2194. 17161244 10.1016/j.jacc.2006.06.002

[6] Hall TS , Hallén J , Krucoff MW , et al. Cardiac troponin I for prediction of clinical outcomes and cardiac function through 3‐month follow‐up after primary percutaneous coronary intervention for ST‐segment elevation myocardial infarction. Am Heart J. 2015;169(2):257‐265. 25641535 10.1016/j.ahj.2014.10.015

[7] Hertzeanu H , Hertzeanu I , Almog C , Zaidman I . Usefulness of myoglobin radioimmunoassay determintion in CCU. Acta Cardiol. 1982;37(4):257‐268. 6981904

[8] Yang Y , Liu J , Zhao F , et al. Analysis of correlation between heart failure in the early stage of acute myocardial infarction and serum pregnancy associated plasma protein‐A, prealbumin, C‐reactive protein, and brain natriuretic peptide levels. Ann Palliat Med. 2022;11(1):26‐34. 35144395 10.21037/apm-21-2993

[9] Oh PC , Choi IS , Ahn T , et al. Predictors of recovery of left ventricular systolic dysfunction after acute myocardial infarction: from the Korean acute myocardial infarction registry and Korean myocardial infarction registry. Korean Circ J. 2013;43(8):527‐533. 24044011 10.4070/kcj.2013.43.8.527 PMC3772297

[10] Ottervanger J . Long‐term recovery of left ventricular function after primary angioplasty for acute myocardial infarction. Eur Heart J. 2001;22(9):785‐790. 11350111 10.1053/euhj.2000.2316

[11] Tasar O , Karabay AK , Oduncu V , Kirma C . Predictors and outcomes of no‐reflow phenomenon in patients with acute ST‐segment elevation myocardial infarction undergoing primary percutaneous coronary intervention. Coron Artery Dis. 2019;30(4):270‐276. 31026233 10.1097/MCA.0000000000000726

[12] Moons KGM , Altman DG , Reitsma JB , et al. Transparent Reporting of a multivariable prediction model for Individual Prognosis or Diagnosis (TRIPOD): explanation and elaboration. Ann Intern Med. 2015;162(1):W1‐W73. 25560730 10.7326/M14-0698

[13] Lei Z , Li B , Li B , Peng W . Predictors and prognostic impact of left ventricular ejection fraction trajectories in patients with ST‐segment elevation myocardial infarction. Aging Clin Exp Res. 2022;34(6):1429‐1438. 35147922 10.1007/s40520-022-02087-y PMC9151544

[14] Thygesen K , Alpert JS , Jaffe AS , et al. Third universal definition of myocardial infarction. J Am Coll Cardiol. 2012;60(16):1581‐1598. 22958960 10.1016/j.jacc.2012.08.001

[15] Authors/Task Force m , Windecker S , Kolh P , et al. 2014 ESC/EACTS guidelines on myocardial revascularization: the Task Force on myocardial revascularization of the European Society of Cardiology (ESC) and the European Association for Cardio‐Thoracic Surgery (EACTS) developed with the special contribution of the European Association of Percutaneous Cardiovascular Interventions (EAPCI). Eur Heart J. 2014;35(37):2541‐2619. 25173339 10.1093/eurheartj/ehu278

[16] Smith Jr. SC , Dove JT , Jacobs AK , et al. ACC/AHA guidelines for percutaneous coronary intervention (revision of the 1993 PTCA guidelines)‐executive summary: a report of the American College of Cardiology/American Heart Association task force on practice guidelines (Committee to revise the 1993 guidelines for percutaneous transluminal coronary angioplasty) endorsed by the Society for Cardiac Angiography and Interventions. Circulation. 2001;103(24):3019‐3041. 11413094 10.1161/01.cir.103.24.3019

[17] Sciagrà R , Parodi G , Migliorini A , Memisha G , Antoniucci D , Pupi A . Evaluation of the influence of age and gender on the relationships between infarct size, infarct severity, and left ventricular ejection fraction in patients successfully treated with primary percutaneous coronary intervention. J Nucl Cardiol. 2010;17(3):444‐449. 20238194 10.1007/s12350-010-9213-7

[18] Serrao GW , Lansky AJ , Mehran R , Stone GW . Predictors of left ventricular ejection fraction improvement after primary stenting in ST‐segment elevation myocardial infarction (from the harmonizing outcomes with revascularization and stents in acute myocardial infarction trial). Am J Cardiol. 2018;121(6):678‐683. 29394998 10.1016/j.amjcard.2017.12.004

[19] Parodi G , Memisha G , Carrabba N , et al. Prevalence, predictors, time course, and long‐term clinical implications of left ventricular functional recovery after mechanical reperfusion for acute myocardial infarction. Am J Cardiol. 2007;100(12):1718‐1722. 18082514 10.1016/j.amjcard.2007.07.022

[20] Bax JJ , Poldermans D , Elhendy A , et al. Improvement of left ventricular ejection fraction, heart failure symptoms and prognosis after revascularization in patients with chronic coronary artery disease and viable myocardium detected by dobutamine stress echocardiography. J Am Coll Cardiol. 1999;34(1):163‐169. 10400006 10.1016/s0735-1097(99)00157-6

[21] Rizzello V . Long term prognostic value of myocardial viability and ischaemia during dobutamine stress echocardiography in patients with ischaemic cardiomyopathy undergoing coronary revascularisation. Heart. 2006;92(2):239‐244. 15814593 10.1136/hrt.2004.055798 PMC1860784

[22] Harrell Jr. FE , Lee KL , Mark DB . Multivariable prognostic models: issues in developing models, evaluating assumptions and adequacy, and measuring and reducing errors. Stat Med. 1996;15(4):361‐387. 8668867 10.1002/(SICI)1097-0258(19960229)15:4<361::AID-SIM168>3.0.CO;2-4

[23] Morris TP , White IR , Royston P . Tuning multiple imputation by predictive mean matching and local residual draws. BMC Med Res Methodol. 2014;14(1):75. 24903709 10.1186/1471-2288-14-75 PMC4051964

[24] Kim JH . Multicollinearity and misleading statistical results. Korean J Anesthesiol. 2019;72(6):558‐569. 31304696 10.4097/kja.19087 PMC6900425

[25] Friedman J , Hastie T , Tibshirani R . Regularization paths for generalized linear models via coordinate descent. J Stat Softw. 2010;33(1):1‐22. 20808728 PMC2929880

[26] Steyerberg EW , Vergouwe Y . Towards better clinical prediction models: seven steps for development and an ABCD for validation. Eur Heart J. 2014;35(29):1925‐1931. 24898551 10.1093/eurheartj/ehu207 PMC4155437

[27] Fitzgerald M , Saville BR , Lewis RJ . Decision curve analysis. JAMA. 2015;313(4):409‐410. 25626037 10.1001/jama.2015.37

[28] Bosch X , Théroux P . Left ventricular ejection fraction to predict early mortality in patients with non‐ST‐segment elevation acute coronary syndromes. Am Heart J. 2005;150(2):215‐220. 16086920 10.1016/j.ahj.2004.09.027

[29] Braunwald E , Rutherford JD . Reversible ischemic left ventricular dysfunction: evidence for the “hibernating myocardium”. J Am Coll Cardiol. 1986;8(6):1467‐1470. 3782649 10.1016/s0735-1097(86)80325-4

[30] Granger CB . Predictors of hospital mortality in the global registry of acute coronary events. Arch Intern Med. 2003;163(19):2345‐2353. 14581255 10.1001/archinte.163.19.2345

[31] Abu‐Assi E , Ferreira‐González I , Ribera A , et al. Do GRACE (Global Registry of Acute Coronary events) risk scores still maintain their performance for predicting mortality in the era of contemporary management of acute coronary syndromes? Am Heart J. 2010;160(5):826‐834. 21095268 10.1016/j.ahj.2010.06.053

[32] Khan MH , Islam MN , Aditya GP , et al. Correlation of troponin‐I level with left ventricular ejection fraction and in‐hospital outcomes after first attack of non‐ST segment elevation myocardial infarction. Mymensingh Med J. 2017;26(4):721‐731. 29208858

[33] Hallen J , Jensen JK , Fagerland MW , Jaffe AS , Atar D . Cardiac troponin I for the prediction of functional recovery and left ventricular remodelling following primary percutaneous coronary intervention for ST‐elevation myocardial infarction. Heart. 2010;96(23):1892‐1897. 21062778 10.1136/hrt.2009.190819

[34] Junior AG , de Almeida TL , Tolouei SEL , Dos Santos AF , Dos Reis Lívero FA . Predictive value of sirtuins in acute myocardial infarction—bridging the bench to the clinical practice. Curr Pharm Des. 2021;27(2):206‐216. 33019924 10.2174/1381612826666201005153848

[35] Tajbakhsh A , Gheibi Hayat SM , Taghizadeh H , et al. COVID‐19 and cardiac injury: clinical manifestations, biomarkers, mechanisms, diagnosis, treatment, and follow up. Expert Rev Anti Infect Ther. 2021;19(3):345‐357. 32921216 10.1080/14787210.2020.1822737

[36] Delanghe JR , Chapelle JP , Vanderschueren SC . Quantitative nephelometric assay for determining myoglobin evaluated. Clin Chem. 1990;36(9):1675‐1678. 2208710

[37] Delanghe JR , De Buyzere ML , Cluyse LP , Thierens HM , Clement DL . Acute myocardial infarction size and myoglobin release into serum. Clin Chem Lab Med. 1992;30(12):823‐830. 10.1515/cclm.1992.30.12.823 1489857

[38] Cheng Z , Shi Y , Peng H , Zhao D , Fan Q , Liu J . Prognostic significance of percutaneous coronary intervention for first acute myocardial infarction with heart failure: five‐year follow‐up results. Cardiol Res Pract. 2022;2022(1):1‐9. 10.1155/2022/5791295 PMC9649329 36386562

[39] Zhou X , Lei M , Zhou D , et al. Clinical factors affecting left ventricular end‐diastolic pressure in patients with acute ST‐segment elevation myocardial infarction. Ann Palliat Med. 2020;9(4):1834‐1840. 32279513 10.21037/apm.2020.03.22

[40] Saito Y , Kobayashi Y . Percutaneous coronary intervention strategies in patients with acute myocardial infarction and multivessel disease: completeness, timing, lesion assessment, and patient status. J Cardiol. 2019;74(2):95‐101. 31053505 10.1016/j.jjcc.2019.04.001

[41] Muller DWM , Topol EJ , Ellis SG , Sigmon KN , Lee K , Califf RM . Multivessel coronary artery disease: a key predictor of short‐term prognosis after reperfusion therapy for acute myocardial infarction. Am Heart J. 1991;121(4 Pt 1):1042‐1049. 1901190 10.1016/0002-8703(91)90661-z

[42] Wu KC , Zerhouni EA , Judd RM , et al. Prognostic significance of microvascular obstruction by magnetic resonance imaging in patients with acute myocardial infarction. Circulation. 1998;97(8):765‐772. 9498540 10.1161/01.cir.97.8.765

[43] Wang L , Liu G , Liu J , Zheng M , Li L . Effects of no‐reflow phenomenon on ventricular systolic synchrony in patients with acute anterior myocardial infarction after percutaneous coronary intervention. Ther Clin Risk Manag. 2016;12(1):1017‐1022. 27445480 10.2147/TCRM.S107808 PMC4928622

[44] Ndrepepa G , Tiroch K , Keta D , et al. Predictive factors and impact of no reflow after primary percutaneous coronary intervention in patients with acute myocardial infarction. Circulation. 2010;3(1):27‐33. 20118156 10.1161/CIRCINTERVENTIONS.109.896225

